# Children's perception about falls and its prevention: a qualitative study from a rural setting in Bangladesh

**DOI:** 10.1186/1471-2458-13-1008

**Published:** 2013-11-06

**Authors:** Salim M Chowdhury, Leif Svanstro, Lars G Horte, Rabiul A Chowdhury, Fazlur Rahman

## Abstract

Background Although the number of smokers has declined in the last decade, smoking is still a major health problem among youngsters and adolescents. For this reason, there is a need for effective smoking prevention programmes targeting primary school children. A web-based computer-tailored feedback programme may be an effective intervention to stimulate youngsters not to start smoking, and increase their knowledge about the adverse effects of smoking and their attitudes and self-efficacy regarding non-smoking. Methods & Design This paper describes the development and evaluation protocol of a web-based out-of-school smoking prevention programme for primary school children (age 10-13 years) entitled 'Fun without Smokes'. It is a transformation of a postal mailed intervention to a web-based intervention. Besides this transformation the effects of prompts will be examined. This web-based intervention will be evaluated in a 2-year cluster randomised controlled trial (c-RCT) with three study arms. An intervention and intervention + prompt condition will be evaluated for effects on smoking behaviour, compared with a no information control condition. Information about pupils' smoking status and other factors related to smoking will be obtained using a web-based questionnaire. After completing the questionnaire pupils in both intervention conditions will receive three computer-tailored feedback letters in their personal e-mail box. Attitudes, social influences and self-efficacy expectations will be the content of these personalised feedback letters. Pupils in the intervention + prompt condition will - in addition to the personalised feedback letters - receive e-mail and SMS messages prompting them to revisit the 'Fun without Smokes' website. The main outcome measures will be ever smoking and the utilisation of the 'Fun without Smokes' website. Measurements will be carried out at baseline, 12 months and 24 months of follow-up. Discussion The present study protocol describes the purpose, intervention design and study protocol of 'Fun without Smokes'. Expectations are that pupils receiving tailored advice will be less likely to smoke after 24 months in contrast to pupils in the control condition. Furthermore, tailored feedback letters and prompting is expected to be more effective than providing tailored feedback letters only. Trial Registration Dutch Trial Register NTR3116

## Ipilimumab efficacy: Optimal dose and schedule

## Pre-publication history

The pre-publication history for this paper can be accessed here:

http://www.biomedcentral.com/1471-2458/13/1008/prepub

